# Primary high-grade urothelial carcinoma of the prostate combined with acinar adenocarcinoma: first rare case report and treatment experience summary

**DOI:** 10.3389/fonc.2025.1649636

**Published:** 2025-10-27

**Authors:** Ting Hu, Yisha Liu, Yu Nie, Chengpeng Gu, Guopeng Wang, Jinze Zhang, Fang Zhou, Shangqing Ren, Dong Wang

**Affiliations:** ^1^ School of Medicine, University of Electronic Science and Technology of China, Chengdu, China; ^2^ Robotic Minimally Invasive Surgery Center, Sichuan Provincial People’s Hospital, School of Medicine, University of Electronic Science and Technology of China, Chengdu, China; ^3^ Department of Pathology, Sichuan Provincial People’s Hospital, School of Medicine, University of Electronic Science and Technology of China, Chengdu, Sichuan, China; ^4^ School of Clinical Medicine, Southwest Medical University, Luzhou, China

**Keywords:** prostate cancer, urothelial carcinoma, acinar adenocarcinoma, immunohistochemistry, robotic surgery, combined therapy

## Abstract

**Introduction:**

Primary high-grade urothelial carcinoma of the prostate (PHUCP) combined with acinar adenocarcinoma is an extremely rare malignancy, with no previously reported cases worldwide. Primary urothelial carcinoma of the prostate (PUCP) accounts for only 1%-4% of all prostate malignancies, and its clinical manifestations often overlap with common prostatic diseases such as benign prostatic hyperplasia (BPH), leading to frequent misdiagnosis. This study presents the first documented case and summarizes the diagnostic and therapeutic approach.

**Case presentation:**

A 61-year-old male patient presented with lower urinary tract symptoms and was initially diagnosed with BPH, later revised to Gleason score 10 prostate adenocarcinoma at external hospitals. Following an innovative port-free single-site robot-assisted radical prostatectomy (pf-ssRARP) at our institution, immunohistochemical analysis (CK7/CK20/GATA3 positive, prostate-specific antigen (PSA) negative) confirmed the diagnosis of PHUCP combined with acinar adenocarcinoma. Postoperatively, a surgery combined with targeted and immunotherapy regimen was initiated: leuprorelin + rezvilutamide for the acinar adenocarcinoma and disitamab vedotin + toripalimab for the urothelial carcinoma. No recurrence or metastasis was observed during the 1-year follow-up period.

**Conclusion:**

This case underscores the diagnostic challenges of prostatic urothelial carcinoma and highlights the importance of immunohistochemistry in cases with normal PSA but rapid progression. The protocol of surgery combined with targeted and immunotherapy offers a new treatment strategy for this rare malignancy. This study provides valuable insights for clinical diagnosis and management.

## Introduction

Prostate cancer is one of the most common malignancies in men, comprising various histological subtypes, predominantly adenocarcinoma (1). Diagnosing rare subtypes like primary urothelial carcinoma of the prostate (PUCP) poses significant diagnostic challenges. PUCP is highly aggressive, representing merely 1%-4% of all prostate cancers (2). Its clinical manifestations often overlap with benign prostatic hyperplasia (BPH) and chronic prostatitis, frequently resulting in diagnostic dilemmas. Imaging studies have limited diagnostic value, and histopathological evaluation supplemented by immunohistochemical analysis constitutes the diagnostic gold standard (3). Due to its rarity, the biological behavior and optimal treatment strategies for PUCP remain incompletely characterized. Moreover, no cases of PUCP combined with acinar adenocarcinoma have been reported previously.

## Case presentation

A 61-year-old male was admitted to our robotic minimally invasive surgery center for surgical evaluation following a transurethral resection of the prostate (TUR-P) performed in September 2023 and a confirmed diagnosis of prostate adenocarcinoma established in February 2024.

The patient initially presented with symptoms including urinary frequency, urgency, dysuria, and interrupted voiding, which did not improve after symptomatic administration of α-blockers and 5α-reductase inhibitors. His total prostate-specific antigen (T-PSA) level was measured at 2.364 ng/mL in September 2023 at an external hospital and, and he underwent TUR-P. Postoperatively, his symptoms temporarily improved, and the pathology suggested BPH, no immunohistochemistry was performed. In December 2023, the patient’s symptoms recurred, accompanied by severe perineal pain and discomfort. In February 2024, he visited another hospital, where abdominal Computed tomography (CT) and prostate Magnetic resonance imaging (MRI) (performed on February 22, 2024) indicated significant prostatic enlargement, with a volume of (6.9 cm × 5.2 cm × 5.7 cm) × 0.52=106.35 mL, suggesting possible prostate malignancy. A prostate biopsy revealed acinar adenocarcinoma (Gleason score: 5 + 5=10; WHO/ISUP grade group: 5). Positron emission tomography - computed tomography (PET-CT) (performed on February 29, 2024) showed no distant metastasis. The patient was subsequently treated with leuprorelin 11.25 mg every 3 months subcutaneously + rezvilutamide 240 mg once daily orally as novel endocrine therapy.

**Figure 1:**
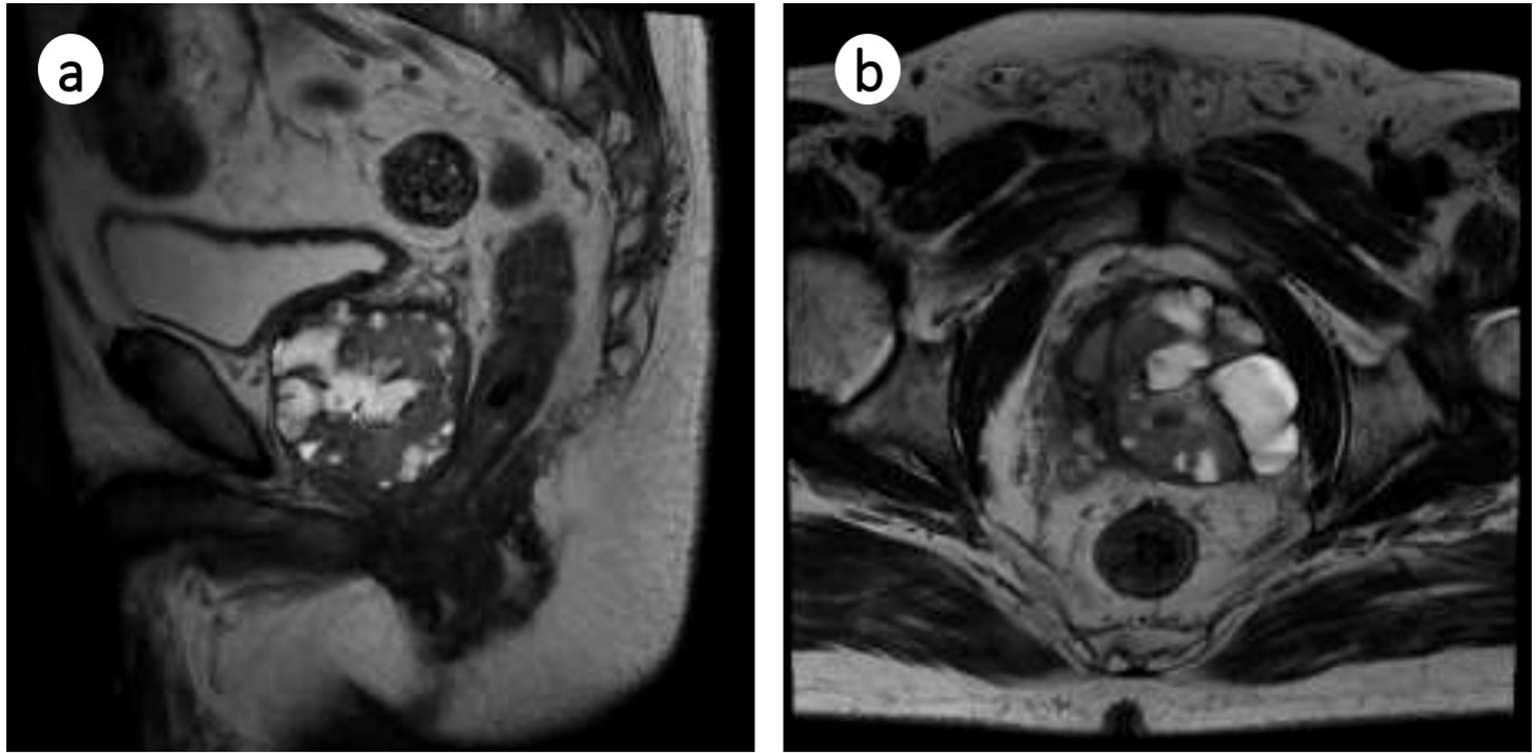
Contrast-enhanced MRI of the prostate. **(a)** Sagittal plane; **(b)** Transverse plane.

In March 2024, the patient was referred to our hospital. Laboratory tests, including complete blood count, liver function, kidney function, coagulation screening, and T-PSA, were all within normal ranges. Enhanced prostate MRI (performed on March 20, 2024) showed a prostate size of (6.9 cm × 5 cm × 5.6 cm) × 0.52=100.46 mL. Multiple patchy and nodular short T2 signals were observed in the prostate parenchyma, with the largest cross-section measuring approximately 40 × 47 × 54 mm. Within these areas, nodular long T2 signals were seen, with heterogeneous enhancement on contrast imaging, high signal on diffusion-weighted imaging (DWI), and low signal on apparent diffusion coefficient (ADC). Involvement of the left seminal vesicle and thickening of the posterior bladder wall were noted, suggesting possible prostate cancer with bladder and left seminal vesicle involvement (**Figure 1**). Single-photon emission computed tomography (SPECT) whole-body bone imaging showed no evidence of metastasis. Cystoscopy revealed no significant abnormalities.

**Figure 2:**
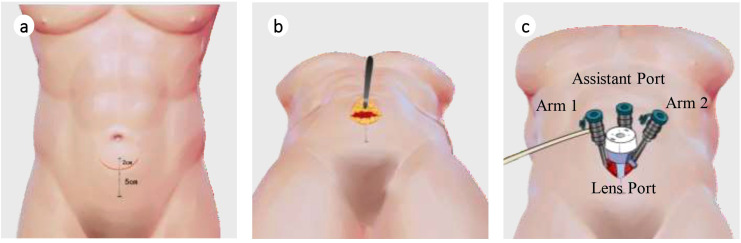
Improved (port-free) single-site robot-assisted laparoscopic radical prostatectomy surgical schematic diagram. **(a)** Determination of marking points; **(b)** Incision of the anterior rectus sheath and separation of the extraperitoneal space; **(c)** Insertion of puncture trocar.

After multidisciplinary discussion, considering the large prostate volume and possible involvement of the bladder and left seminal vesicle, the surgical difficulty was assessed as high. The patient subsequently underwent an innovative pf-ssRARP under general anesthesia (**Figure 2**). This technique utilizes the anatomical features of the anterolateral abdominal wall and the elasticity of the skin flap to complete single-port surgical procedures that would otherwise require a port, through a small curved incision (5 cm). Intraoperatively, the prostate volume was approximately 120 cm³, adherent to the pelvis, with visible scars from the previous TUR-P. The median lobe protruded into the bladder by 0.8 cm, and both seminal vesicles were firm and tightly adherent to surrounding tissues. Tumor infiltration was evident in the left lobe and apex of the prostate. The surgery was successful, and the patient recovered well.

**Figure 3:**
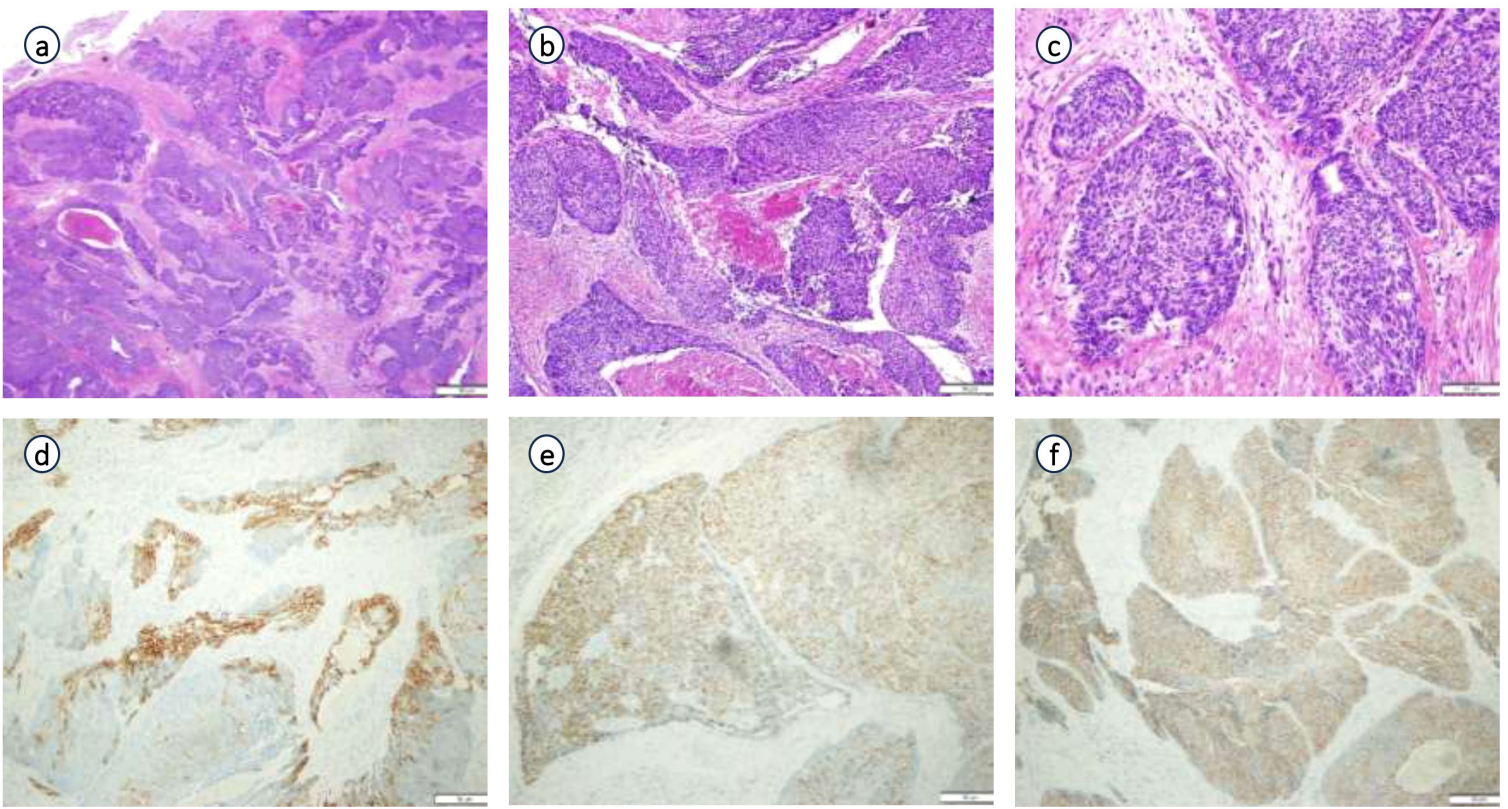
Pathological examination and immunohistochemistry of surgically resected specimen: **(a)** (×20 HE), **(b)** (×100 HE), **(c)** (×200 HE) of pathological examination; **(d)** (CK 7 partial +); **(e)** (GATA3 partial +); **(f)** (P540S +) of immunohistochemistry.

**Figure 4:**
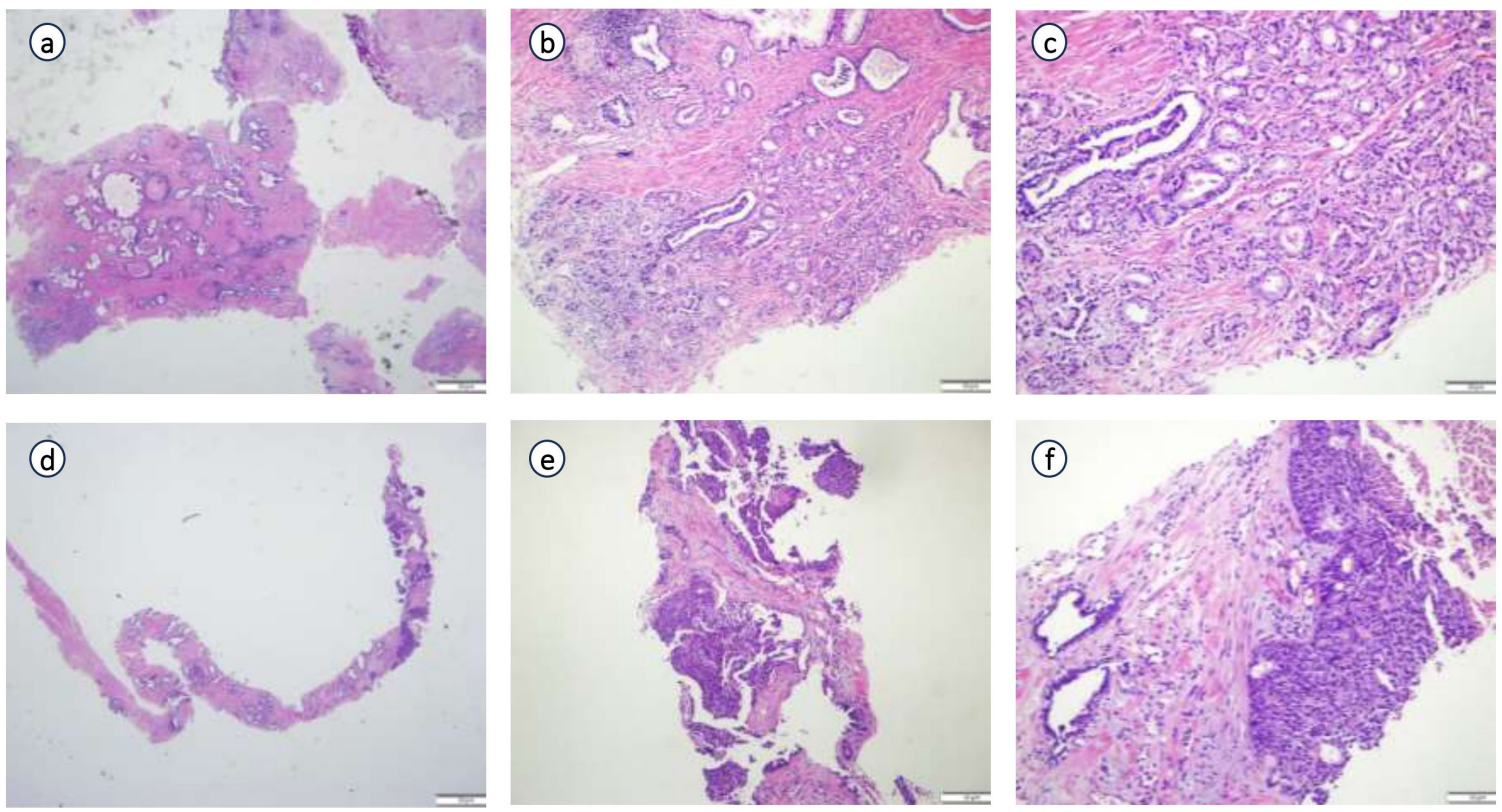
Pathological consultation of previous specimens in our hospital. **(a)** (×20 HE), **(b)** (×100 HE), **(c)** (×200 HE): Results from pathological consultation of transurethral resection of prostate (TURP) specimens performed at an external hospital; **(d)** (x 20 HE), **(e)** (×100 HE), **(f)** (x200 HE): Results from pathological consultation of prostate needle biopsy specimens performed at an external hospital.

Postoperative pathology findings: Tumor infiltration was present at the upper margin of the specimen and in the left seminal vesicle, but not at the lower margin, in the right seminal vesicle, at the distal ends of both vas deferens, or in the surrounding lymph nodes. Immunohistochemistry (**Figure 3**) showed the following results: tumor cells were partially positive for CK, focally positive for CK7, individually positive for CK20, partially positive for GATA3, individually positive for P40 and P63, HER2 (1+), focally positive for 34βE12, negative for CgA, INSM1, NKX3.1, PSA, and Syn, positive for P504S, BRG, and INI-1, and negative for CD34 and SALL4. The Ki67 proliferation index was approximately 40%. Combined with morphological and immunophenotypic features, the findings indicated high-grade invasive urothelial carcinoma with focal glandular differentiation. Pathological consultation of previous specimens at our hospital revealed that the initial TUR-P specimen was consistent with acinar adenocarcinoma (Gleason score: 3 + 4=7; WHO/ISUP grade group: 2), while the prostate biopsy specimen suggested urothelial carcinoma (**Figure 4**). Integrating all pathological results, the final diagnosis was PHUCP combined with acinar adenocarcinoma. According to the eighth edition of the UICC, the patient’s final TNM staging is T3bN0M0 stage III.

The patient was initially diagnosed with BPH and later puncture suggested high-grade prostate adenocarcinoma. The diagnosis was finally clarified by obtaining a complete pathology specimen through surgery and several pathology consultations.

Following confirmation of the diagnosis, and considering the patient’s mixed histopathological subtypes and positive surgical margins, a targeted combined immunotherapy regimen was initiated. The existing protocol established by the external hospital—consisting of leucovorin (11.25 mg administered every 3 months) and revelutamide (240 mg administered daily)—was continued for the adenoalveolar adenocarcinoma component. Concurrently, combination immunotherapy targeting the uroepithelial carcinoma component was implemented, comprising vedicilizumab (120 mg every 2 weeks) plus terteplizumab (240 mg every 3 weeks) for 6 cycles. Follow-up PET-CT (performed on August 28, 2024) and cystoscopy performed five months post-treatment revealed no abnormalities. Furthermore, surveillance at 1 year post-surgery revealed no evidence of disease recurrence or metastasis.

## Discussion and conclusions

PUCP is a rare malignant tumor that accounts for 1%-4% of prostate cancers (1). Previous studies [e.g., Shen et al. (2), Palou et al. (3)] have shown that PUCP is usually a single invasive tumor, and the disease has a high rate of initial diagnosis, which is often misdiagnosed as BPH or prostate adenocarcinoma, and most of them have progressed to pT3+ stage by the time of diagnosis (4). This is the first reported case of PHUCP coexisting with adenocarcinoma, suggesting that mixed histologic types need to be considered for atypical clinical manifestations.

Histopathological examination reveals that PUCP demonstrates characteristic features of high-grade urothelial carcinoma, exhibiting marked cellular atypia and abundant mitotic activity. Immunohistochemical analysis serves as a pivotal diagnostic tool, with PUCP typically exhibiting positive staining for GATA3, CK7, CK20, and P63 - markers that reliably identify tumors of urothelial lineage (5). In contrast, acinar adenocarcinoma predominantly expresses PSA and NKX3.1 while remaining negative for GATA3 and CK20 in most instances (6). Of particular interest in our case was the discordance between a normal serum PSA level (2.364 ng/mL) and an exceptionally high Gleason score (10), which initially obscured the urothelial carcinoma component. This observation corroborates the findings of Chuang et al. (7), who established that PSA-negative but CK7+/CK20+ tumors strongly suggest urothelial differentiation. Additionally, P504S typically shows negative expression in pure prostatic urothelial carcinoma (8).

The diagnostic evaluation of PUCP necessitates not only differentiation from conventional prostate adenocarcinoma but also assessment for potential coexistence of both tumor types. The accurate diagnosis of mixed tumors presents considerable challenges; however, comprehensive immunohistochemical panels significantly enhance diagnostic precision. The urothelial component can be confirmed by positive staining for GATA3 and p63, while PSA and NKX3.1 reliably identify the adenocarcinoma component. Importantly, the immunohistochemical profile of P504S positivity coupled with P63 and 34βE12 negativity provides a distinctive pattern for differentiating urothelial carcinoma from prostate adenocarcinoma in coexisting cases (9). It should be noted that certain high-grade prostate adenocarcinomas may demonstrate diminished expression or complete loss of prostate-specific markers, necessitating correlation with clinical PSA levels and radiological findings for accurate interpretation.

Initial clinical presentation typically includes lower urinary tract symptoms such as urinary frequency, urgency, dysuria, voiding difficulty, and intermittent stream, which are nonspecific and often lead to diagnostic confusion with other prostatic disorders (10). Definitive diagnosis mandates cystoscopic evaluation and transurethral resection to exclude primary bladder tumor involvement of the prostate (11). In the current case, several diagnostic challenges were encountered: 1) Excessive reliance on PSA screening; 2) Ambiguous pathological diagnosis following initial TURP at a referring institution; and 3) Significant morphological overlap between high-grade adenocarcinoma and urothelial carcinoma components. The diagnosis was ultimately confirmed through extensive immunohistochemical profiling and multidisciplinary pathological consultation.

The management of this rare tumor combination demands innovative therapeutic strategies. We employed a multimodal approach integrating surgical intervention with targeted and immunotherapeutic modalities:

Radical prostatectomy remains the cornerstone of treatment for localized disease. Therefore, we performed pf-ssRARP on the patient. While surgical resection alone can be effective, review of published cases suggests that combination therapy incorporating chemotherapy may offer superior clinical outcomes. Han et al. (12) reported sustained disease-free survival at 1-year follow-up in a PUCP patient treated with surgery and adjuvant chemotherapy.

From a pharmacological perspective, PUCP exhibits distinct characteristics from other prostate malignancies, particularly its hormone-independent nature which renders androgen deprivation therapy ineffective. The coexistence of conventional adenocarcinoma with positive surgical margins in our case necessitated divergent treatment strategies for the hormone-sensitive and chemotherapy-resistant components. For the adenocarcinoma component, we maintained the referring institution’s protocol of revelutamide (240 mg daily) combined with leuprolide, based on the established efficacy and safety profile demonstrated in the CHART study (13). For the PUCP component, we adopted treatment principles from advanced bladder cancer management (14). While gemcitabine-cisplatin (GC) combination chemotherapy represents first-line therapy, concerns regarding toxicity and the complex tumor biology prompted utilization of novel ADC+PD1 combination immunotherapy (vedicilizumab + terteplizumab), which has demonstrated both safety and efficacy in recent clinical trials (15).

In conclusion, we report for the first time a case of primary high-grade uroepithelial carcinoma of the prostate combined with follicular adenocarcinoma. This case brings important insights: first, the diagnosis of primary uroepithelial carcinoma of the prostate needs to be considered as a possible diagnosis for PSA-negative but high Gleason score prostate tumors with large tumor size, and immunohistochemistry is mandatory to avoid misdiagnosis; second, the mixed histologic type (urothelial carcinoma + adenocarcinoma) requires well-designed multimodal treatment with multiple lines of surgery combined with endocrine therapy and targeted immunotherapy, as explored in this case. therapy, which was explored as a feasible treatment option in this case; third, pf-ssRARP is a viable surgical option for large-volume tumors with multiple advantages. good results at 1-year follow-up support this comprehensive treatment strategy. Future studies should explore biomarkers such as HER2 to further optimize targeted therapies for this rare tumor combination.

## Data Availability

The raw data supporting the conclusions of this article will be made available by the authors, without undue reservation.
